# Investigation of antimicrobial susceptibility and genetic diversity among *Staphylococcus pseudintermedius* isolated from dogs in Rio de Janeiro

**DOI:** 10.1038/s41598-023-47549-z

**Published:** 2023-11-18

**Authors:** Izabel Mello Teixeira, Yasmim de Moraes Assumpção, Ana Clara Cabral Paletta, Louise Aguiar, Luciana Guimarães, Isabella Thomaz da Silva, Marina F. Côrtes, Ana Maria Nunes Botelho, Lauren Hubert Jaeger, Renata Fernandes Ferreira, Eliane de Oliveira Ferreira, Bruno Penna

**Affiliations:** 1 Laboratório de Biologia de Anaeróbios, Departamento Microbiologia Médica, IMPPG, UFRJ, Rio de Janeiro, Brazil; 2 Laboratório de Cocos Gram Positivos, Departamento de Microbiologia e Parasitologia, UFF, Rio de Janeiro, Brazil; 3 Laboratório de Células-Tronco e Parasitologia Molecular, Departamento de Ciências Farmacêuticas, UFJF, Juiz de Fora, Brazil; 4 Universidade de Vassouras, Vassouras, RJ Brazil; 5 Laboratório de Investigação Médica, Instituto de Medicina Tropical, USP, São Paulo, Brazil

**Keywords:** Microbiology, Antimicrobials, Bacteriology, Clinical microbiology, Microbial genetics

## Abstract

*Staphylococcus pseudintermedius* is an opportunistic pathogen causing a variety of infections that are difficult to treat, especially because of the development of antimicrobial resistance. It has a clonal distribution around the world. To have a better understanding of the MRSP population, we search the presence of MRSP in colonized or infected dogs. Samples from 99 dogs with infections and 35 from asymptomatic dogs were collected. Isolates were identified by mass spectrometry and Multiplex-PCR. The *mecA* gene was confirmed by conventional PCR. MRSP strains were analyzed by whole-genome sequencing. 75 *S. pseudintermedius* were identified, most from infection cases. The species were isolated from 70 out of the 135 dogs. Penicillin and Trimethoprim/Sulfamethoxazole presented higher resistance rates. Forty-seven strains were classified as multi-drug resistant (MDR), and were more isolated from dogs with infection (P < 0.05). Eighteen samples were classified as MRSP, representing 24.0% of the population. Six of 16 MRSP sequenced samples belonged to the world spread clone ST71; others belonged to unknown clones. Most samples carried the *SCCmec* type IIIA. Twenty-one different genetic resistance determinants were found among MRPS strains. MRSP is circulating among infected and colonized dogs in Rio de Janeiro, Brazil.

## Introduction

*S. pseudintermedius*, first described in 2005^1^, is a significant component of the cutaneous microbiome of healthy dogs^2^. It is also the leading cause of canine skin, ear, and urinary tract infections and post-surgical wounds^3,4^. Although it is not typically isolated from humans, cross-species transmissions have already been documented^5^. Nonetheless this zoonotic potential of *S. pseudintermedius* strains can not be neglected since these samples may be misidentified as *Staphylococcus aureus*^6^. Close contact between pets and their owners can facilitate zoonotic transmission^7^.

Over the past few years, studies have increasingly reported Methicillin-resistant *Staphylococcus pseudintermedius* (MRSP) infections. MRSP is often resistant to a range of other antimicrobials and, in some cases, considered a multi-resistant strain^8^. The emergence of multidrug-resistant bacteria (MDR) such as MRSP strains poses a threat to antimicrobial therapy in veterinary medicine due to limited treatment options. Previous studies have demonstrated an outstanding clonal diversity among *S. pseudintermedius*, with more than 1400 sequence types (STs) reported. The most successful lineages reported are ST71, ST68, and ST45^9^. The ST71 lineage initially identified in Europe is the most widespread^10^. In Brazil, very few studies regarding MRSP epidemiology are available. Penna and co-workers demonstrated the spread of the ST71 lineage in Rio de Janeiro state^11^. More recently, Viegas and co-workers also identified new clones circulating in Belo Horizonte. Furthermore, *SCCmec* and/or other antimicrobial resistance genes can be transferred between different staphylococci, which can lead to serious public health issues, especially when involving MRSP and methicillin-resistant *S. aureus* (MRSA).

Studies on the dynamics of the population and molecular epidemiology of infections caused by multi-resistant staphylococci in companion animals can generate critical data to guide public health programs. The health and welfare of dogs and other companion animals and the close interactions humans have with them necessitate a deeper understanding of the bacterial pathogens they harbor. Thus, we evaluated *S. pseudintermedius* distribution among infected and colonized dogs. We also analyzed the antimicrobial resistance profile of the studied isolates, and clonal distribution among MRSP.

## Results

### *Staphylococcus pseudintermedius* isolation

Seventy-five samples of *S. pseudintermedius* were obtained from 134 dogs (75/134 dogs or 52.2%). Most samples—53/75 or 70.6%—were isolated from infection cases. The other 22 samples (29.3%) were isolated from colonization sites. Among infection samples, the majority—43/53 or 77.4%—were obtained from canine pyoderma. Most of the isolates prevenient from asymptomatic dogs were obtained from the perineum (12/22 or 54.5% of the samples). *S. pseudintermedius* were proportionally more isolated from infection sites (51 dogs) than from colonization sites (19 dogs) in the dogs (p-value < 0.05). Table 1 depicts results regarding *S. pseudintermedius* distribution.

**Table 1:** *S. pseudintermedius* isolation distribution among infection sites and colonization sites of dogs from Rio de Janeiro (2016–2017).

Source	No of isolates
Pyoderma	43
Otitis	8
Urinary tract infection	2
Nasal fossa	9
Perineum	12
Oral cavity	1
Total	75

### Phenotypic resistance profile

Among *S. pseudintermedius* strains, the drugs presenting the higher resistance rates in the disk diffusion test were penicillin (76.0%; 57/75) and Trimethoprim/Sulfamethoxazole (60.0%; 45/75), followed by Erythromycin (54.7%; 41/75), Tetracycline (53.3%; 40/75), and Ciprofloxacin (49.3%; 37/75). Three antimicrobials had the lower resistance rates: Rifampicin, Doxycycline (3/75; 4.0% respectively), and Nitrofurantoin (2/75; 2.7%). Regarding resistance to multiple antimicrobials, 47 of the 75 strains were classified as multi-drug resistant (MDR), representing 63.5% of the samples. MDR samples were significantly more isolated from infected dogs than colonized dogs (P < 0.05). Graphic 1 shows the results of antimicrobial resistance rates of *S. pseudintermedius.*

Oxacillin resistance was found in 14 out of the 75 samples by disc diffusion method, but 18 samples were genotypically classified as MRSP by the presence of the *mecA* gene, representing 24.0% of the isolates. Seventeen dogs were carrying these MRSP strains, representing a total of 12.7% (17/134). Regarding origin, dogs with pyoderma or otitis presented most MRSP samples (16/18). Only two MRSP strains were from asymptomatic dogs. Graphic 1 shows the results of antimicrobial resistance rates of all samples and among MRSP strains.

After sequencing, the processes failed in one of the MRSP samples (LB1619), resulting in 25 samples with their genome sequenced. 16 MRSP samples, and 9 MSSP. Regarding the antimicrobial resistance, 21 resistance determinants were found among MRSP and MSSP strains. These genes encoded resistance to a wide range of antimicrobials, such as Beta lactams, Aminoglycosides, Macrolides, Fosfomycin, Chloramphenicol, Quinolones, and other drugs. Five of these 21 genes (23.8%) were identified in all samples. These genes were: *blaZ* (Betla lactamic), *sdrM, norA* (Quinolones)*, fosB* (Phosfomycin). The gene *ykkcd* encoding a small multi-drug efflux pump, conferring resistance to Phenicol, Tetracyclines, and Aminoglycosides, was also found in all samples. The *sepA* gene, which confers resistance to disinfecting agents, was found in 92% of the samples (23/25). The genes *aac(6')-aph(2'')*, which confers resistance to aminoglycosides, and *dfrG*, responsible for resistance to Trimethopim, were found in 84% of the samples (21/25).

Other genes that also showed a high prevalence in the population were: genes related to resistance to aminoglycosides such as *aph(3')-III, ant(6)-Ia, sat4* found in 80% of the samples (20/25); the *erm(B)* gene responsible for the MLSb phenotype, also found in 80% of the samples. The *aad(6)* gene, which confers resistance to aminoglycosides, was found in 68% of the samples (17/25), and the *tet(M)* gene, which provides resistance to tetracycline, was found in 56% of the samples (14/25).

Other genes had a smaller distribution than those mentioned above, but still with a considerable prevalence. The *cat(pC221)* gene, for example, which confers resistance to chloramphenicol, was found in 36% of the samples (9/25), and was more found among MSSP samples.The *qacG* gene, which confers resistance to chlorhexidine and disinfectant agents, was found in 32% of the samples. (9/25). The genes with the lowest prevalence in the population were: *tet(K)*, which confers resistance to tetracyclines, found in only two samples (8%) of MRSP, and the *qacJ* gene, which confers resistance to disinfecting agents, found in only one (4%). A noteworthy fact is that samples belonging to ST2283/SCCmec V had the same resistance profile with the same genetic determinants of resistance. The same pattern was not observed in ST71/SCCmecIIIA strains. Supplementary file S1 reunites information regarding the phenotypic and genotypic resistance profile and antimicrobial resistance genes from MRSP strains.

### MLST and *SCCmec* typing

**Figure 1:**
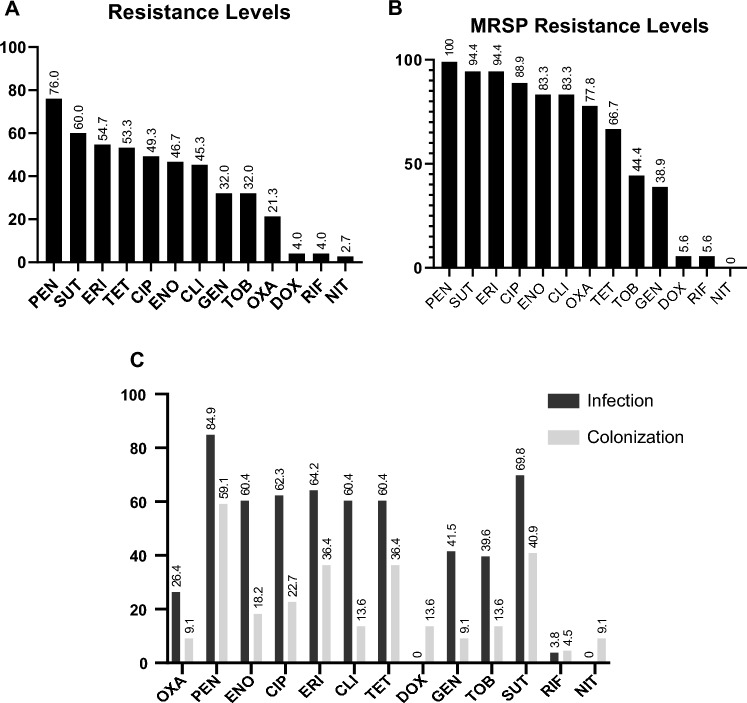
Resistance rates of MSSP and MRSP strains isolated from dogs with infection and colonized. (**A**) Indicates resistance level of all *S. pseudintermedius* strains (MSSP and MRSP). (**B**) Indicates resistance levels of MRSP strains alone. (**C**) Indicates resistance level of MSSP and MRSP strains separated by site of origin (infection samples x colonization samples). *OXA* oxacillin, *PEN* penicillin, *ENO* enrofloxacin, *CIP* ciprofloxacin, *ERI* erythromycin, *CLI* clindamycin, *TET* tetracycline, *DOX* doxycycline, *GEN* gentamicin, *TOB* tobramycin, *SUT* trimethoprim/sulfamethoxazole, *RIF* rifampicin, *NIT* nitrofurantoin.

**Figure 2:**
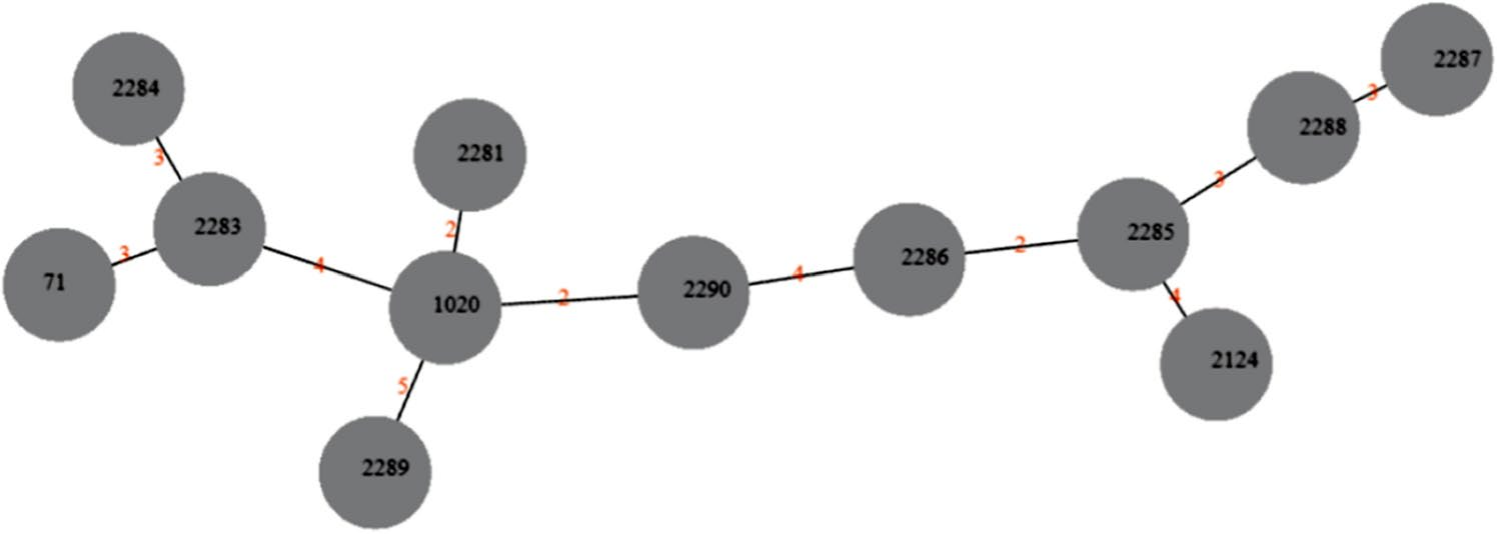
goeBURST analyses indicating relations between the new STs (gray circles) found in this study. Numbers in red indicates absolute distance between the types.

**Figure 3:**
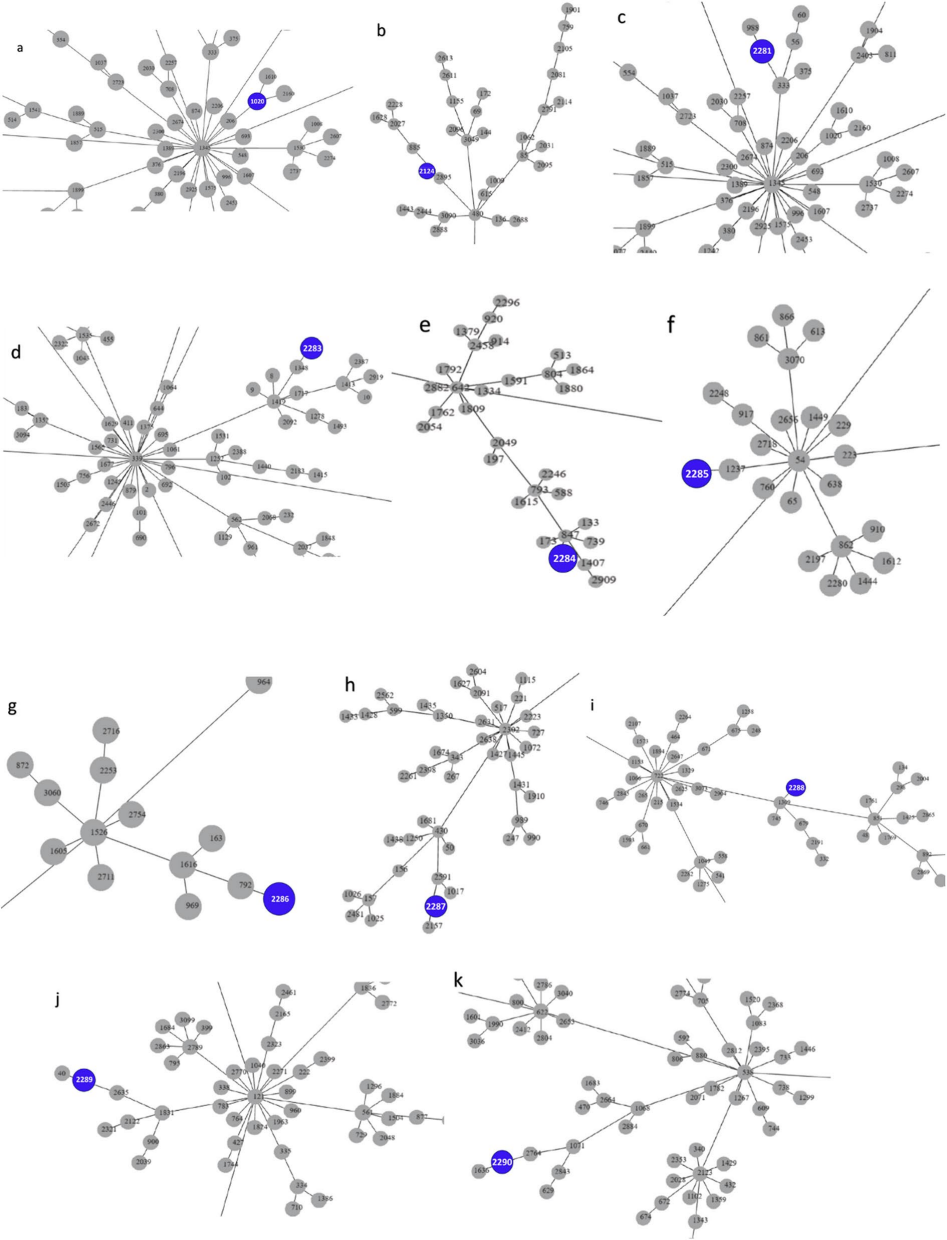
goeBURST analyses showing relations between new strain types found in this study and other strains around the world (**a**) Shows ST1020 belonging to CC1345. (**b**) Shown ST2124 belonging to CC480. (**c**) Shows ST2281 belonging to CC1345. (**d**) Shows ST2283 belonging to CC339. (**e**) Shows ST2284 belonging to CC642. (**f**) Shows ST2285 belonging to CC54. (**g**) Shows ST2286 belonging to CC1526. (**h**) Shows ST2287 belonging to CC2302. (**i**) Shows ST2288 belonging to CC722. (**j**) Shows ST2289 belonging to CC121. (**k**) Shows ST2290 belonging to CC538.

**Figure 4:**
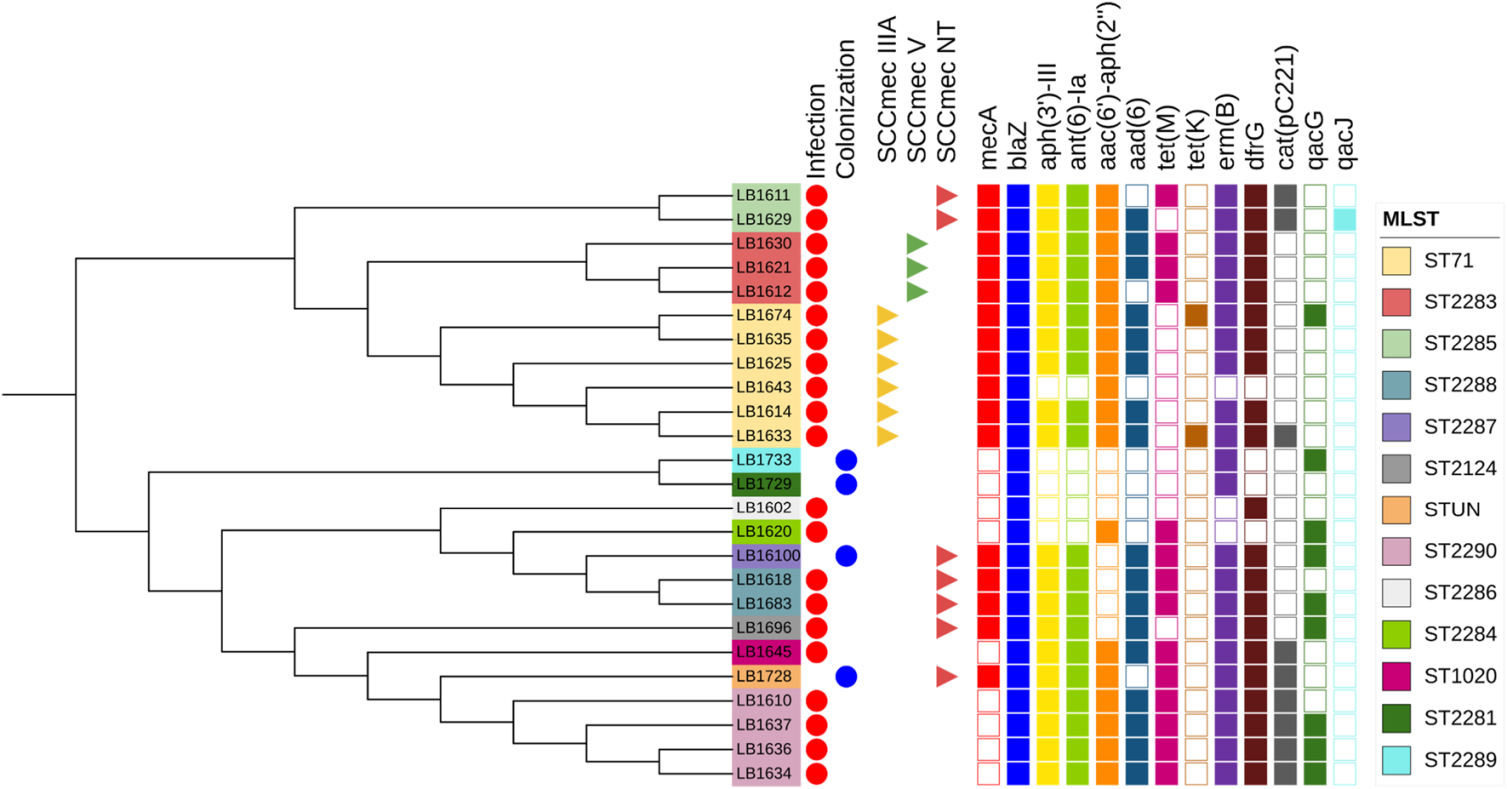
Phylogenetic Tree with information regarding sample origin (represented by red and blue dots), MLST (diferent colours in the legend on the right), SCCmec type (represented by yellow, green and red triagels) and prevalence of genetic determinants of resistance (coloured squares on the right) in the *S. pseudintermedius* population sequenced.

Six of the 16 MRSP samples belonged to the world-spread clone ST71*-SCCmec*IIIa, representing 37.5% of the MRSP strains. This clone was isolated from six different dogs (7.1%). Other samples belonged to new STs. Three were ST2283-SCC*mec*V (20.0%), two were ST2285-SCC*mec*NT, two were ST2288-SCC*mec*NT (13.3% each), one ST2124-SCC*mec*NT, and one ST2287-SCC*mec*NT (6.7% each). One sample (1728), provenient from a colonization site (nasal fossa), could not have its ST typed, being characterized as "Unknown ST". Regarding *SCCmec* typing, most samples belonged to *SCCmec* type IIIA (6/15 or 40.0%). Three samples were from *SCCmec* type V (3/15 or 20.0%). The remaining samples could not have their *SCCmec* typed by the chosen method. In the group of MSSP strains, six different types of clones were also observed. The most prevalent was clone ST2290—4 out of 9 samples or 44.4%. The other clones found had only one representant (11.1%) each, and were: ST2286, ST2284, ST1020, ST2281, ST2289. Phylogenetic tree depicted in Fig. 1 represents relations stablished among MRSP and MSSP samples, as well as sample origin, MLST, SCCmec type and prevalence of genetic determinants of resistance. Figure 2 represents relations between the new MLST types found in this study. goeBURST analyses indicated that none of the new ST types belonged to world spread clonal complexes, besides ST71, belonging to CC71 (Fig. 3). Figure 4 contains information regarding sample origin, MLST, SCCmec type and prevalence of genetic determinants of resistance in the *S. pseudintermedius* population sequenced.

## Discussion

*S. pseudintermedius* is the most isolated species from dogs (representing up to 90% of all isolated staphylococci), both from the asymptomatic dogs and in cases of canine opportunistic infections, such as otitis and pyoderma^12^. In this study, in both symptomatic and asymptomatic dogs, *S. pseudintermedius* was recovered in most samples. From a one-health perspective, this could represent a potential zoonotic risk. Other researchers have already studied *S. pseudintermedius* isolation in a similar dog population. Studies in Australia^13^ and Japan^14^ evaluating canine pyoderma *S. pseudintermedius* was found on 88.8% (24/27) and 76.4% (94/123) of the dogs, respectively. When evaluating asymptomatic dogs, Bean and Wigmore (2016)^15^ found that sites where *S. pseudintermedius* was most isolated, were the perineum (61.5% or isolated from 72/117 dogs), the oral cavity (60.7%) and nasal cavities (44.4%). In our study, the perineum was also the site with higher isolation of *S. pseudintermedius*. Approximately ten years ago our group already reported *Staphylococcus pseudintermedius* as a leading cause of canine pyoderma and otitis externa^16,17^, and one of the major causes of canine UTI^18^. And only recently another report from Penna and co-workers reported that *S. pseudintermedius* was recovered from 58.5% of canine samples from a similar population^11^.

A worrying fact is the increasing emergence of resistant strains over the years. Several studies have already reported resistant samples of veterinary origin^19–23^. High resistance rates to penicillin (76.0%) were not surprising since practitioners widely use them in human and veterinary medicine. Previous studies also identified high levels of resistance to penicillinase label penicillin^17^. Not surprisingly, other antimicrobials with high resistance rates were erythromycin, Trimethoprim/Sulfamethoxazole, and Tetracycline. Our group has already reported multiple levels of resistance to these drugs in a similar dog population^11,17^. Other studies in Brazil also found high resistance rates to commonly used antimicrobials. Viegas and coworkers (2022)^24^, recently also found resistance among erythromycin (32.1%), Trimethoprim/Sulfamethoxazole (44.3%), and Tetracycline (43.5%). Another study conducted in Thailand found resistance rates of 28.3% for Trimethoprim/Sulfamethoxazole and 37.7% for Tetracycline^25^.

In our study, MRSP could be recovered from 18 samples, representing 22.6% of the *S. pseudintermedius* population. Seventeen dogs were carrying these strains. Some studies also found considerable rates of MRSP samples like ours. The prevalence of MRSP isolation was similar in studies carried out in Argentina^26,27^ and Germany^28^. Still, another study conducted in Japan with a similar population found an MRSP rate of 22.3% (21/94)^14^. These studies observed that strains of MRSP are generally more isolated from infected sites, such as pyoderma and otitis. Noteworthy is the high prevalence of multi-drug resistant strains (63.5%). A study conducted by our group with samples collected around 2011–2012 also identified a MDR rate of 49% among *S. pseudintermedius* strains^11^. On the other hand, the same study only found 6.1% of MRSPs among infected and colonized dogs indicating a significant increase of MRSP isolation (P < 0.05).

Regarding *SCCmec* typing, nine out of 15 samples could have their chromosomal cassette typed. The majority (6/15) belonged to *SCCmec* type IIIA and the rest to type V. A recent study conducted in the United States also identified *SCCmec* type III as the main cassette in MRSP population, followed by types IV and V^29^. Another study in Australia found *SCCmec* type V the most prevalent, followed by III, IV, and II^30^. *SCCmec* type III is frequently associated with hospital-acquired *Staphylococcus aureus* (HA-MRSA) and carries a set of other resistance genes^31^, representing a challenge in terms of antimicrobial therapy, especially in veterinary medicine. The *SCCmec* could not be typed in six MRSP isolates. Currently, there are 14 types of *SCCmec* described and numerous subtypes, most of which are well-characterized on *S. aureus* species. There is a lack of studies about *SCCmec* diversity in non-*aureus* species^32^. Novel *SCCmec* types have already been identified in *S. pseudintermedius* but are unavailable in databases^33,34^.

*S. pseudintermedius* has a worldwide distribution, with a great diversity of molecular types and clonal complexes. Recent studies have focused on investigating the main circulating sequence types worldwide^8,10,29,30,32,35^. The main circulating clone worldwide seems to be ST71, the most isolated in North America, Europe, and Oceania. In our study, ST71 was also the most isolated in the MRSP population, representing 40% of the strains (6/15). Despite the lack of studies regarding the molecular epidemiology of *S. pesudintermedius* in South America, three other Brazilian studies have also identified ST71 circulation in MRSP populations in the country, and in one of them, it was the most prevalent clone^24,36^. The study conducted by our group with canine samples obtained among 2011–2012 only identified clone ST71 among 17 MRSP isolates^11^. Few epidemiological studies on MRSP epidemiology are available, especially in our country. A study from Argentina with infected dogs did not detect ST71 MRSP among 10 MRSP detected^26^. Other clones detected in this study (ST2283; ST2285; ST2288; ST2124; ST2287) were newly identified clones, never isolated in other countries around the world, nor in Brazil or other South American countries (https://pubmlst.org/organisms/staphylococcus-pseudintermedius). Therefore, this study provides new information about *S. pseudintermedius* types and its antimicrobial resistance pattern. All new STs were resistant to at least 7 different classes of non-beta-lactam antimicrobials, and almost all of them (only ST2124 and ST2287 were found in only one dog) were isolated from more than one animal. This could indicate local dispersion of new STs of MRSPs and highlights the need for more studies in Brazil to better understand the evolution of these new lineages

Finally, this study could investigate the genotypic antimicrobial resistance profile in MRSP strains. The set of genes found were resemblant to those found in other studies with similar populations^29,30,32^, with genes conferring resistance to a set of antimicrobials such as aminoglycosides, tetracyclines, Chloramphenicol, Quinolones, Trimethoprim, and other classes. Noteworthy all samples in this study presented the gene *fosB* conferring resistance to Fosfomycin, a fact not observed elsewhere^30,32^. MRSP samples also presented a diversity of genes conferring resistance to disinfectants and antiseptic agents (sepA, qacG, qacJ). Specifically, qac genes (quaternary ammonium compounds) can be responsible for chlorhexidine resistance, a usual biocide used to treat skin infections in dogs. Genetic determinants of biocide tolerance in *Staphylococcus* are a growing concern^34^.

The presence of high rates of resistance to antimicrobial agents and the carriage of different resistance genes is a worrying factor among MRSP samples, also classified as MDR strains. It poses an extra challenge in treating topic infections in dogs, remaining only limited treatment options. In this study, samples were resistant to antibiotics commonly used in veterinary practice (such as gentamicin and ciprofloxacin)^37^. The first-choice antimicrobials for treating pyoderma in dogs are Cephalexin and Trimethoprim/Sulfamethoxazole, both with high resistance rates in general and MRSP populations. Almost all samples also carried the *dfrG* gene, which encodes resistance to Trimethoprim/Sulfamethoxazole. In this scenario, first-choice treatments are unfeasible.

Enrofloxacin, which is among second-choice antimicrobial agents used for pyoderma treatment, also had high resistance rates in our study (46.7%). Moreover, among the MRSP population, genetic determinants of Quinolone resistance were found (*norA*; *sdrM*). Gentamicin usually is another second-choice antimicrobial for canine pyoderma treatment. In our study, resistance to aminoglycosides also had considerable rates (>40%). All MRSP samples carried at least one aminoglycoside resistance gene. With these results, we can infer that *S. pseudintermedius*, especially MRSP strains, are resistant to commonly used antimicrobials in veterinary clinics' routines.

In conclusion, our study could recover a considerable amount of MRSP strains from infected and colonized dogs. Most of these strains belonged to the world-spread clone ST71 and carried other resistance genes besides *blaZ* and *mecA*. Zoonotic transmission has been documented a few times despite being a canine-related species. Under the concept of One Health, this species may also represent a risk for humans, similar to other species within the genus. The close social interaction between companion animals and humans can favor the exchange of strains. Understanding the bacterial populations that inhabit companion animals is critical to restraining their spread and securing the health of pets and humans.

## Methods

### Animals included in the study

We investigated 134 adult dogs (> 1 year) who attended a veterinary clinic (VetCare) in the neighborhood of Laranjeiras in Rio de Janeiro State, Brazil. Dogs were divided into infected or asymptomatic groups. The first group, of infected dogs, included 99 dogs presenting signs of pyoderma, otitis, or urinary tract infection (UTI). Pyoderma symptoms included papules, pustules, circular crusts, dry or flaky patches of skin. Otitis symptoms were redness of the skin, swelling, scratching, increased discharge, and scaly skin. Lastly, signs of UTI included hematuria and dysuria; urinalysis results including red blood cell counts > 5 per field and proteinuria; and > 10^3^ colony-forming units of bacteria per mL of urine at the first plating. The second group, of asymptomatic dogs, included 35 dogs with no clinical signs of infection (asymptomatic dogs) in routine care. All samples were collected between July 2016 and June 2017. There was no distinction made between race or gender for inclusion criteria. The collections and use of dog samples was approved by the Animal Use Ethics Committee of Fluminense Federal University (protocol number 1008), and all experiments were performed in accordance with relevant guidelines and regulations. Also, this study is in accordance with ARRIVE guidelines.

### Sample collection and processing

All canine pyoderma or otitis samples were collected using sterile cotton swabs (Copan Diagnostic, Italy). In animals affected by pyoderma, samples were collected from unruptured pustules; in otitis, samples were collected from the auricular canal. In canine UTI, 5 ml of urine was collected. In asymptomatic dogs, swabs were collected from the nasal fossa, perineum, and oral cavity. Samples were seeded into Salt Mannitol Agar (KASVI, Italy), incubated aerobically at 37 °C for 24 h, and then stocked in a − 80 freezer.

### Mass spectrometry of MALDI-TOF type

All isolates were firstly identified by mass spectrometry of MALDI-TOF type (Matrix-assisted laser desorption ionization-time of flight) (Biotyper-Bruker) according to manufacturer's orientation. Isolated colonies were first transferred, in duplicate, with the aid of a sterile loop, to the MALDI-TOF identification plate. Subsequently, 1µl of 70% formic acid and 1 µl of matrix (αcyanic acid 4-hydroxycinnamic acid) was added to each well. The plate was left on the bench to dry and the plate was inserted into the device for reading. Identification criteria values used were those recommended by the manufacturer, using a score ≥ 2000 for identification at the species level. This score is given by Bruker's Biotyper program (version 3.1) by comparing the spectra from the equipment's database and the spectrum obtained from sample analysis, ranging from 0.0 to 3.0, with 0.0 to 1.699 not presenting a reliable identification, from 1.7 to 1.999 representing probable identification of the genus, from 2.0 to 2.299 representing confirmation of the genus and probable identification of the species and from 2.3 to 3.0 representing a high probability of identification of the species. Device calibration was performed with a standardized sample of Escherichia coli DH5a and the mass spectra followed the following parameters: laser frequency 20 Hz; voltage of ionic sources 1 and 2, 20 kV and 18.6 kV respectively; molecular weight range 2000–20,000 Da.

### Antimicrobial susceptibility Test

Strains were analyzed for antimicrobial resistance by the disc-diffusion method and interpreted by recommendations of the Clinical and Laboratory Standards Institute (CLSI 2020 and CLSI VET 2013). Antimicrobial agents tested were cefoxitin (30 μg), ciprofloxacin (5 μg), clindamycin (2 μg), doxycycline (30 μg), enrofloxacin (5 μg), erythromycin (15 μg), gentamicin (10 μg), nitrofurantoin (10 μg), penicillin G (10 IU), rifampicin (30 μg), sulfamethoxazole/trimethoprim (23.75 μg/1.25 μg), tetracycline (30 μg) and tobramycin (10 μg). As recommended, we used a strain of *S. aureus* (ATCC25923) as a positive control for the test. Strains resistant to 3 or more antimicrobial classes were classified as Multi-Drug Resistant (MDR)^38^.

### PCR detection of the *nuc* and *mec*A gene

DNA extraction was accomplished by thermal lysis using chelex (Bio-Rad) as described by Walsh and co-workers^39^. The extracted DNA was quantified using Quantus Fluorometer (Promega, Madison, EUA) according to the manufacturer's instructions. For both PCR, we used Promega Kit (GoTaq® G2 DNA Polymerase), and the concentrations of the reagents were: 5X Green Reaction Buffer, 0.2 mM of dNTPs, 1.5 mM of MgCl_2_, ten pmol of each primer (Table 1), 2U of GoTaq, and 100 ng of DNA template in a total reaction volume of 25 µl. All isolates belonging to the *Staphylococcus intermedius* group were confirmed by a PCR targeting the thermonuclease gene (*nuc*), according to Sasaki et al., 2010^40^. This PCR consisted of an initial denaturation step at 95 °C for two minutes, followed by 30 cycles at 95 °C for 30 s, 56 °C for 35 s and 72 °C for one minute, and a final extension step of one cycle at 72 °C for two minutes. A sample of *S. pseudintermedius* (ED99) was used as a positive control for all reactions. For the detection of the *mecA* gene, PCR was conducted according to the recommendations of Zhang et al.^41^. Promega Kit (GoTaq® G2 DNA Polymerase) was used with the same reagent’s concentrations (5X Green Reaction Buffer, 0.2 mM of dNTPs, 1.5 mM of MgCl_2_, ten pmol of each primer). This PCR consisted of an initial denaturation step at 94 °C for 5 min followed by 30 cycles of 94 °C for 1 min, 50 °C for 1 min, and 72 °C for 2 min, ending with a final extension step at 72 °C for 10 min. A sample of *Staphylococcus aureus* (USA 100) previously identified as having de *mecA* gene, was used as a positive control for the *mecA* gene. Both amplifications (*nuc and mecA*) were performed in a Veriti thermocycler (Applied Biosystems, California, USA). Both reaction products (*nuc* and *mecA*) were visualized under ultraviolet light stained with Gel Red (Invitrogen) using A 1 Kb (Invitrogen) molecular size marker.

### Whole-genome sequencing and genome assembly

For whole-genome sequencing (WGS), we analyzed only one MRSP strain from each dog, and we selected 9 random MSSP samples, from 9 different dogs for WGS and comparison, resulting in a total of 26 samples sent to WGS. Samples were cultured in Tryptone Soy Agar (KASVI, Italy) and incubated aerobically at 37 °C for 24 h. DNA extraction was conducted using Wizard Genomic DNA Kit (Promega, Madison, USA) according to manufacturer's instructions. The extracted DNA was quantified using Quantus Fluorometer (Promega, Madison, EUA) according to the manufacturer's instructions.

Library preparation was performed using Illumina DNA prep (M) Tagmentation (96 samples) and Nextera DNA CD index (96 indexes, 96 samples) (Illumina, San Diego, California, USA), according to the manufacturer’s instructions. Libraries were subsequently quantified using Qubit and dsDNA HS-kit (Thermo Fisher, Waltham, Massachusetts, USA). Finally, the sample was loaded on a HiSeq2500 system and ran for 201 cycles (PE125), pair-end (500 bp library) using HiSeq Rapid SBS Kit v2 chemistry. The quality of the generated files was evaluated by FASTQC v.0.11.3. The reads were trimmed using Trimmomatic v. 0.33. The assembly of the genome and ordering of the contigs were performed using the programs BWA MEM and Samtools using Reference strain HSP125 (accession number CP066708.1). Genome annotation was performed using prokka v1.14.6. MLST typing was performed in silico using the MLST tool from the Center for genomic epidemiology (CGE) (https://cge.food.dtu.dk/services/MLST/) and the PubMLST tool in Galaxy (https://usegalaxy.org/). SCC*mec* elements were identified using the CGE tool SCCmecFinder (https://cge.food.dtu.dk/services/SCCmecFinder/). The genetic determinants of resistance were searched using two tools: ResFinder from CGE (https://cge.food.dtu.dk/services/ResFinder/) and The Comprehensive Antibiotic Resistance Database—CARD (https://card.mcmaster.ca/home). Parameters used in the analysis of MLST, SCCmec, and research of resistance genes were those pre-established by the tools. For the search of resistance determinants nucleotide analysis were used. For the Single Nucleotide Polymorphisms (SNP) analysis, the fastq files were mapped to the reference with default parameters then the contigs of the pairs were aligned using bowtie2 and the variable sites were visualized using Seaview version 5.0.4 (Galtier et al., 1996) and REALPHY (Bertels et al., 2014) tool where multiple alignments were recreated using PhyML considering bootstrap of 500 replications for the reconstruction of the phylogenetic tree. The tool used to annotate and edit the phylogenetic tree was iTOL v.6.

### Statistical analysis

Statistical Analysis was conducted using GraphPad PRISM (8.0.1) software, and the methodology used was Fisher's Exact Test. Values of P < 0.005 were considered statistically significant.

### Supplementary Information

Supplementary Information.

## Data Availability

The datasets generated during and/or analysed during the current study are are deposited in NCBI under the submission number SUB12820865 and will be made publicly available once the ID is accepted. All data are available from the corresponding author on reasonable request.
