# Supplementary material for: Investigation of antimicrobial susceptibility and genetic diversity among Staphylococcus pseudintermedius isolated from dogs in Rio de Janeiro
Source: Sci Rep. 2023 Nov 18;13:20219. doi: 10.1038/s41598-023-47549-z (PMC10657392; doi:10.1038/s41598-023-47549-z)
Supplement: Supplementary file 1 — Supplementary Information. [file 41598_2023_47549_MOESM1_ESM.docx]

| **Sample** | **Origin** | **SCCmec** | **Phenotypic Resistance Profile** | **Genotypic Resistance profile** |
| --- | --- | --- | --- | --- |
| LB1611 | P | NT | oxa,pen, eno, cip, eri, cli, tet, gen, tob, sut | *blaZ; mecA; aph(3')-III; ant(6)-Ia; aac(6')-aph(2''); sat4; tet(M); erm(B); dfrG; fosB; cat(pC221); sdrM; norA; ykkcd* |
| LB1612 | P | V | oxa,pen, eno, cip, eri, cli, tet, tob, sut | *blaZ; mecA; aph(3')-III; ant(6)-Ia; aac(6')-aph(2''); sat4; tet(M); erm(B); dfrG; fosB; sdrM; norA; sepA; ykkcd* |
| LB1614 | P | IIIA | oxa, pen, eri, cli, tet, sut | *blaZ; mecA; aph(3')-III; ant(6)-Ia; aac(6')-aph(2''); aad(6); sat4; erm(B); dfrG; fosB; sdrM; norA; sepA; ykkcd* |
| LB1618 | O | NT | oxa, pen, eno, cip, eri, cli, tet, sut | *blaZ; mecA; aph(3')-III; ant(6)-Ia; aad(6); sat4; tet(M); erm(B); dfrG; fosB; sdrM; norA; sepA; ykkcd* |
| LB1621 | P | V | oxa, pen, eno, cip, eri, cli, tet, sut | *blaZ; mecA; aph(3')-III; ant(6)-Ia; aac(6')-aph(2''); aad(6); sat4; tet(M); erm(B); dfrG; fosB; sdrM; norA; sepA; ykkcd* |
| LB1625 | P | IIIA | pen, eno, cip, eri, cli, gen, tob, sut | *blaZ; mecA; aph(3')-III; ant(6)-Ia; aac(6')-aph(2''); aad(6); sat4; erm(B); dfrG; fosB; sdrM; norA; sepA; ykkcd* |
| LB1629 | P | NT | oxa, pen, eno, cip, eri, cli, tet, gen, tob, sut | *blaZ; mecA; aph(3')-III; ant(6)-Ia; aac(6')-aph(2''); aad(6); sat4; erm(B); dfrG; fosB; cat(pC221); sdrM; norA; sepA; qacJ; ykkcd* |
| LB1630 | P | V | oxa, pen, eno, cip, eri, cli, tet, gen, tob, sut | *blaZ; mecA; aph(3')-III; ant(6)-Ia; aac(6')-aph(2''); aad(6); sat4; tet(M); erm(B); dfrG; fosB; sdrM; norA; sepA; ykkcd* |
| LB1633 | P | IIIA | oxa, pen, eno, cip, eri, cli, tet, gen, tob, sut | *blaZ; mecA; aph(3')-III; ant(6)-Ia; aac(6')-aph(2''); aad(6); sat4; tet(K); erm(B); dfrG; fosB; cat(pC221); sdrM; norA; sepA; ykkcd* |
| LB1635 | P | IIIA | oxa, pen, eno, cip, eri, cli, gen, tob, sut | *blaZ; mecA; aph(3')-III; ant(6)-Ia; aac(6')-aph(2''); aad(6); sat4; erm(B); dfrG; fosB; sdrM; norA; sepA; ykkcd* |
| LB1643 | P | IIIA | oxa, pen, eno, cip | *blaZ, mecA, aac(6')-aph(2''), fosB, sdrM, norA, sepA, ykkcd* |
| LB1674 | O | IIIA | oxa, pen, eno, cip, eri, cli, tet, sut | *blaZ; mecA; aph(3')-III; ant(6)-Ia; aad(6); sat4; tet(K); erm(B); dfrG; fosB; sdrM; norA; qacG; sepA; ykkcd* |
| LB1683 | P | NT | oxa, pen, eno, cip, eri, cli, tet, sut | *blaZ; mecA; aph(3')-III; ant(6)-Ia; aac(6')-aph(2''); aad(6); sat4; tet(M); erm(B); dfrG; fosB; sdrM; norA; qacG; sepA; ykkcd* |
| LB1696 | P | NT | pen, eri, gen, tob, sut | *blaZ; mecA; aph(3')-III; ant(6)-Ia; aac(6')-aph(2''); aad(6); sat4; erm(B); dfrG; fosB; sdrM; norA; qacG; sepA; ykkcd* |
| LB16100 | NF | NT | oxa, pen, eno, cip, eri, cli, sut | *blaZ; mecA; aph(3')-III; ant(6)-Ia; aad(6); sat4; tet(M); erm(B); dfrG; fosB; sdrM; norA; sepA; ykkcd* |
| LB1728 | PR | NT | pen, eno, cip, eri, tet, dox, sut | *blaZ; mecA; aph(3')-III; ant(6)-Ia; aac(6')-aph(2''); sat4; tet(M); erm(B); dfrG; fosB; sdrM; norA; qacG; sepA; ykkcd* |
| LB1602 | P | X | pen, sut | *blaZ; dfrG* |
| LB1610 | P | X | 0 | *blaZ; aph(3')-III; ant(6)-Ia; aac(6')-aph(2''); tet(M); dfrG; erm(B); cat(pC221)* |
| LB1620 | P | X | pen, tet, gen, tob | *blaZ; aac(6')-aph(2''); tet(M)* |
| LB1634 | P | X | pen, eno, cip, eri, cli, tet, sut | *blaZ; aph(3')-III; ant(6)-Ia; aac(6')-aph(2''); tet(M); dfrG; erm(B); cat(pC221); qacG* |
| LB1636 | P | X | pen, eno, cip, eri, cli, tet, gen, tob, sut | *blaZ; aph(3')-III; ant(6)-Ia; aac(6')-aph(2''); tet(M); dfrG; erm(B); cat(pC221); qacG* |
| LB1637 | P | X | pen, eno, cip, eri, cli, tet, sut | *blaZ; aph(3')-III; ant(6)-Ia; aac(6')-aph(2''); tet(M); dfrG; erm(B); cat(pC221)* |
| LB1645 | O | X | pen, eno, cip, eri, cli, tet, gen, tob, sut, rit | *blaZ; aph(3')-III; ant(6)-Ia; aac(6')-aph(2''); tet(M); dfrG; erm(B); cat(pC221)* |
| LB1729 | NF | X | pen | *blaZ* |
| LB1733 | NF | X | 0 | *qacG* |

**Suplementary File 1:** Information regarding origin, SCCmec type, and phenotypic and genotypic resistance profile of MRSP and MSSP samples of *S. pseudintermedius*. P = pyoderma; O = Otitis; NF = Nasal Fossa; PR = perineum.
